# Editorial: Special Issue on the Epidemiology of Human Papilloma Virus-Associated Oropharyngeal Squamous Cell Carcinoma

**DOI:** 10.3390/cancers15184608

**Published:** 2023-09-18

**Authors:** John F. Mills, Neil P. Monaghan, Shaun A. Nguyen, John Pang, Ameya A. Asarkar, Cherie-Ann O. Nathan

**Affiliations:** 1 Department of Otolaryngology-Head and Neck Surgery, Medical University of South Carolina, Charleston, SC 29425, USA; jom344@musc.edu (J.F.M.); monaghne@musc.edu (N.P.M.); 2 Department of Otolaryngology-Head and Neck Surgery, Louisiana State University Health Sciences Center, Shreveport, LA 71103, USA; john.pang@lsuhs.edu (J.P.); ameya.asarkar@lsuhs.edu (A.A.A.); cnatha@lsuhsc.edu (C.-A.O.N.)

In this Special Issue of *Cancers*, the role of oncogenic human papilloma virus (HPV) with oropharyngeal squamous cell carcinoma is explored. The articles cover a wide range of topics including diagnostic considerations, principles of treatment, prevention, and future aims and directions. We hope this Special Issue will inform current clinicians and researchers about this increasingly common cancer.

Head and neck cancer is the seventh most common type of cancer in the world, with squamous cell carcinoma comprising over 90% of cases [1]. The incidence of these cancers associated with HPV has recently increased among young adults under the age of 45 years [2,3]. In patients with newly diagnosed oropharyngeal cancer, the identification of high-risk HPV strains from the primary tumor or cervical node metastasis is recommended [4]. This can be achieved through detection of E6 or E7 mRNA from fine-needle aspiration specimens or immunohistochemical staining assessing for p16 expression [1]. New detection methods have emerged, including the evaluation of circulating DNA (also referred to as the liquid biopsy), in HPV-related oropharyngeal cancer. The study by Adilbay in this Special Issue describes a greater than 90% sensitivity and nearly 100% specificity in diagnosis with the use of circulating DNA, while also proposing a use for the determination of HPV status in equivocal cases [5]. Additionally, the possibility of replacing surveillance imaging with circulating DNA sampling is explored.

There are over 200 different HPV subtypes, with type 16 accounting for at least 80% of associated oropharyngeal squamous cell carcinomas [6]. However, there may be interactions between variants of HPV that could have clinical implications. In the article by Al-Soneidar, preliminary evidence suggests that the carcinogenic effects of HPV16 may be weakened by a co-infection with beta HPV, whereas coinfection with gamma HPV could strengthen the carcinogenic effect [7]. These cutaneous HPV variants have been identified in skin warts and the oral cavity, and the interplay between variants may prove to be an important factor in future diagnosis, staging, and treatment options.

Once the diagnosis and stage of disease is established, a multimodality approach is initiated, with surgery being the primary treatment strategy in most cases. Depending on the extent of the primary tumor, en-bloc surgical resection of the primary site with clear pathological margins, while maintaining functional and aesthetic outcomes, is recommended [8]. New surgical approaches have been implemented, with one being robotic-assisted surgery. This was first used in the 1970s as a way of providing surgery to astronauts in space [9] and was adopted for transoral use in the early 2000s [10]. While the anatomic subsite of head and neck cancers is an important general consideration, it is also important to recognize potential challenges of anatomic sites within the oropharyngeal designation. The article by Poupore evaluates the efficacy of transoral robotic surgery of the tonsil versus the base of the tongue by evaluating margin status, recurrence, and complication rates [11]. While there was a higher rate of positive margins in the base of the tongue, there was no difference in locoregional or metastatic recurrence rates between these subsites. Despite this single difference, there were no other differences in outcomes when using this surgical modality between the subsites, including post-operative hemorrhage rates. This surgical approach remains a viable treatment option for the oropharyngeal region.

In addition to surgical treatment, radiotherapy used as adjuvant therapy is recommended if the pathological specimen displays adverse features [12]. Soliman et al. demonstrate the benefit of improved survival in patients with early-stage HPV-associated oropharyngeal cancer who were found to have adverse pathological features [13]. Importantly, the authors identified nearly 13% of patients with adverse features who did not undergo adjuvant radiotherapy, with an increase in the rate of these patients over the study period. This underscores the importance of adherence to established National Comprehensive Cancer Network-recommended guidelines for adjuvant therapy.

In addition to the success of first-line treatment, there is always a need for new therapies with reliable efficacy demonstrated in clinical trials. One of the more exciting therapies has been the use of immunotherapy in patients with recurrent or metastatic disease. The checkpoint inhibitor drugs that target the PD-1/PD-L1 pathway, such as nivolumab, have been beneficial in improving overall survival in patients with HPV-associated cancer [14]. However, one of the important considerations in clinical trials is that the sample population may not be representative of the general population [15]. Gordis et al. demonstrate that the demographic profiles of those enrolled in clinical trials and those in the general population are similar [16].

Salvage surgery exists in case of recurrent or persistent disease after initial definitive treatment [17,18]. Unfortunately, these patients have historically poor survival outcomes, and the impact of HPV positivity has not been well established due to a lack of reported survival characteristics in patients that undergo salvage surgery (see the article by Taniguchi in this issue) [19]. The need for future research into survival measures in these patients is needed.

Primary prevention has immense potential for reducing disease burden. The HPV vaccination has been shown to be highly effective in reducing the development of cervical precancers in women [20], and it has been shown to reduce the rates of HPV-positive infections in those already testing positive [21]. While the vaccination remains the only primary prevention for HPV-associated oropharyngeal squamous cell carcinoma, there is a remarkably low rate of the population that receive the full vaccination series, with significant disparities existing among men and Black Americans (Khalil et al.) [22]. Perhaps the most urgent goal moving forward would be to encourage vaccination, through strengthening the patient–provider relationship and community education on the risks of HPV, and subsequently, HPV-associated oropharyngeal squamous cell carcinoma.
